# Response of Soil Fungal Community Structure to Long-Term Continuous Soybean Cropping

**DOI:** 10.3389/fmicb.2018.03316

**Published:** 2019-01-09

**Authors:** Hang Liu, Fengjuan Pan, Xiaozeng Han, Fengbin Song, Zhiming Zhang, Jun Yan, Yanli Xu

**Affiliations:** ^1^National Observation Station of Hailun Agro-ecology System, Key Laboratory of Mollisols Agroecology, Northeast Institute of Geography and Agroecology, Chinese Academy of Sciences, Harbin, China; ^2^University of Chinese Academy of Sciences, Beijing, China

**Keywords:** continuous soybean cropping, fungal community structure, pathogenic fungi, beneficial fungi, fungal relative abundance

## Abstract

Long-term continuous soybean cropping can lead to the aggravation of soil fungal disease. However, the manner in which the fungal community and functional groups of fungi are affected by continuous soybean cropping remains unclear. We investigated the fungal abundance, composition and diversity during soybean rotation (RS), 2-year (SS) and long-term (CS) continuous soybean cropping systems using quantitative real-time PCR and high-throughput sequencing. The results showed that the fungal abundance was significantly higher in CS than in SS and RS. CS altered the fungal composition. Compared with RS, SS had an increase of 29 and a decrease of 12 genera in fungal relative abundance, and CS increased 38 and decreased 17 genera. The Shannon index was significantly higher in CS and SS than in RS. The result of principal coordinate analysis (PCoA) showed that CS and SS grouped together and were clearly separated from RS on the PCoA1. A total of 32 features accounted for the differences in fungal composition across RS, SS, and CS. The relative abundance of 10 potentially pathogenic and 10 potentially beneficial fungi changed, and most of their relative abundances dramatically increased in SS and CS compared with RS. Our study indicated that CS results in selective stress on pathogenic and beneficial fungi and causes the development of the fungal community structure that is antagonistic to plant health.

## Introduction

Soil microbes play a vital role in the agroecosystem, because they are involved in nutrient cycling, organic matter decomposition and soil-borne disease development (Heijden et al., 2008). Continuous or monoculture cropping, a practice of repeated cultivation of the same crop for multiple years, is a common agricultural practice over the world. During long-term continuous soybean (*Glycine max* Merr.) cropping, the crop roots secrete the same exudates for a long time, and some of the crop root secretions such as phenolic acids can alter the microbial community structure and result in an increase in the abundance of pathogenic fungi (Guo et al., 2011; Wang et al., 2013). Due to the industrial demand and the cultivation habits of farmers, CS has increased in recent years which often causes significant decline in soybean yields and quality as a consequence of severe occurrences of soil-borne diseases (Liu and Herbert, 2002). Therefore, understanding how CS affects the soil fungal community may help in developing practices to relieve soil-borne diseases and obstacles associated with soybean cultivation.

Soil fungi can be designated as harmful or beneficial groups based on their functions in agroecosystem. Some fungi can infect crops and cause plant diseases, while others can suppress pathogenic fungi to the plant, benefit plant growth or decompose plant residues (Setälä and McLean, 2004). Continuous cropping can change the fungal composition; for instance, continuous soybean cropping increased the population of *Fusarium* and tended to increase the susceptibility to root rot (Jie et al., 2015). Compared with soybean and maize (*Zea mays* L.) rotations, CS decreased the populations of *Trichoderma* and *Gliocladium* that may play roles in the biocontrol of soil-borne pathogens (Meriles et al., 2009; Pérez-Brandán et al., 2014). Previous studies in the same fields found that the populations of *Fusarium* spp. and *Heterodera glycines* during 20-year continuous soybean cropping were significantly lower than during 3-year continuous soybean cropping, while the populations of *Pochonia chlamydosporia, Paecilomyces lilacinus*, and *Pseudomonas fluorescens* were higher than those in the 3-year continuous soybean cropping and the rotation of soybean/maize/wheat (*Triticum aestivum* L.) (Wei et al., 2015). The populations of *Fusarium equiseti, Pochonia chlamydosporia* and *Purpureocillium lilacinum* that were isolated from parasitized cysts during 21-year continuous soybean cropping were distinctly larger than those during 3-year continuous soybean cropping (Song et al., 2017). The percentage of *Pochonia chlamydosporia* and *Purpureocillium lilacinum* that were isolated from the cysts of the soybean cyst nematodes during the 23-year continuous soybean cropping were notably higher than that during the SS (Song et al., 2016). However, the general changes of the fungal composition and potentially pathogenic and beneficial fungi have yet to be addressed during CS.

Recently, a growing number of studies have focused on the effect of long-term continuous cropping on the soil microbial community structure using different techniques (Vargas et al., 2011; Li et al., 2014b; Wu et al., 2015). A denaturing gradient gel electrophoresis (DGGE) analysis showed an obvious difference in the DGGE bands between the continuous and the rotation soybean cropping systems (Li et al., 2010). The phospholipids fatty acid (PLFA) signatures indicated that the microbial community structure of the rhizosphere changed within the span of continuous soybean cropping, and the daidzein and genistein released by the soybeans had a significant influence on the fungal community (Guo et al., 2011). The terminal restriction fragment length polymorphism (T-RFLP) profiles demonstrated clear differences in the relative abundance, logarithmic transformation and Bray-Curtis dissimilarity matrix of the fungal community during a three-year gradient continuous *Rehmannia glutinosa* cropping (Wu et al., 2015). Using 454 pyrosequencing, Li et al. (2014b) found that continuous peanut (*Arachis hypogaea* Linn) cropping caused a significant accumulation of the pathogenic fungi *Fusarium oxysporum, Phoma* and *Bionectria ochroleuca*, while the relative abundance of the beneficial fungi *Trichoderma* and *Mortierella elongate* was significantly decreased. Because of the limitation of techniques in previous studies, only certain dominant microbial groups were detected in continuous soybean cropping. It is necessary to conduct systematic and comprehensive research on the change of the soil fungal community structure in CS.

With the development of high throughput sequencing technology, abundant information of soil microbial structure, diversity and function can be obtained, which can be used to thoroughly investigate changes in soil fungal community. Thus, in this study, the main hypotheses were that the CS altered the fungal community structure and functions. We used high-throughput sequencing methods to examine the fungal composition and functions during RS, 2-year short-term and 27-year long-term continuous soybean cropping systems in northeast China. The objectives of this study were (1) to reveal the fungal abundance and community composition in the three different soybean cropping systems; (2) to compare the changes of fungal diversity among the three different soybean cropping systems, and (3) to investigate the changes of potentially pathogenic and beneficial fungi in the three different soybean cropping systems. Understanding the effects of continuous soybean cropping on fungal community will provide theoretical guidance for development of improved agricultural management strategies.

## Materials and Methods

### Study Site Description and Soil Sample Collection

Soil samples were collected from a long-term field experiment at the Hailun Experimental Station of Agricultural Ecology, Heilongjiang Province in northeast China (47°26′N, 126°38′E) and in the central region of the Mollisol in Northeast China. The mean annual precipitation was approximately 500–600 mm, with approximately 80% occurring from May to September, and the annual cumulative temperature (≥10°C) ranged from 2400 to 2500°C-days. The long-term field experiment was established in 1991. Before field experiment establishing, a rotation of maize-soybean-wheat crops was applied in this field. In order to homogenize soil chemical fertility across the experimental plots, wheat was grown without fertilization in 1990. In 1991, the large triplicate plots were subdivided into 77-m^2^ plots to establish a long-term cropping system including continuous cropping of wheat, maize and soybean, and wheat-maize-RS. The basal fertilizers N and P fertilizers were applied as urea and ammonium phosphate. There were 65.5 kg N ha^-1^ and 30.1 kg P ha^-1^ applied for maize, 27.0 kg N ha^-1^ and 30.1 kg P ha^-1^ for soybean, and 92.4 kg N ha^-1^ and 16.9 kg P ha^-1^ for wheat. An additional 65.5 kg N ha^-1^ was applied at the booting stage of maize. Details of experimental design and management history have been described in a previous study (Qiao et al., 2015).

A subset of the parent experiment consisting of three soybean cropping systems was selected for present study, including (1) soybean phase of a 3-year rotation of wheat, maize and soybean (RS), (2) short-term continuous cropping on the second soybean year of a 3-year rotation of 1-year wheat and 2-year soybean (SS), and (3) CS.

Soil samples were collected on July 11, 2017 (at the flowering stage) with three replicates. Soybean plants were excavated from the soil, and the soil mass surrounding the roots was collected as bulk soil. A random collection of 10 soybean root soils constituted a soil sample. The soil samples were placed into separate sterile bags and transported to the laboratory, where the plant roots, residues and gravel were moved, and the samples were thoroughly homogenized through a 2-mm soil sieve. The soil samples were stored at -80°C for subsequent soil DNA extraction.

### Soil DNA Extraction

Microbial DNA was extracted from 0.5 g samples using an E.Z.N.A.^®^ soil DNA Kit (Omega Bio-tek, Norcross, GA, United States) according to the manufacturer’s instructions. The final DNA concentration and purification were determined using a NanoDrop 2000 UV-Vis spectrophotometer (Thermo Scientific, Wilmington, DE, United States), and the DNA quality was checked using 1% agarose gel electrophoresis.

### Quantitative Real-Time PCR (Q-PCR)

The fungal ITS region of the rRNA gene copy number for all samples was determined in triplicate using Q-PCR in an ABI 7500 Real-Time PCR System (Applied Biosystems, Carlsbad, CA, United States) with the primer set ITS1F/ITS2R (Adams et al., 2013). Each PCR reaction contained 16.5 μL of AceQ^®^ SYBR Green qPCR Master Mix (2X), 0.8 μL of 5 μM forward and reverse primers (each) and 2.0 μL of template DNA. The PCR samples were subsequently incubated at 95°C for 5 min, followed by 40 cycles of 5 s at 95°C, 30 s for annealing at 50°C, and 40 s for elongation at 72°C. Negative controls consisted of all the reagents with sterilized water instead of soil DNA. The threshold cycle (Ct) was obtained from triplicate samples and averaged. The copy number of the fungal ITS genes was calculated using a regression equation to convert the cycle threshold (Ct) value to the known number of copies in the standard curves.

### Illumina MiSeq Sequencing

The ITS rRNA genes were amplified using the primers ITS1F (5′-CTTGGTCATTTAGAGGAAGTAA-3′) and ITS2R (5′-GCTGCGTTCTTCATC GATGC-3′) (Adams et al., 2013) in a thermocycler PCR system (GeneAmp 9700, ABI, Foster, CA, United States). The PCR reactions were conducted using the following program: 3 min of denaturation at 95°C, 35 cycles of 30 s at 95°C, 30 s for annealing at 55°C, and 45 s for elongation at 72°C, and a final extension at 72°C for 10 min. The PCR reactions were performed in triplicate with a 20 μL mixture containing 4 μL of 5 × FastPfu Buffer, 2 μL of 2.5 mM dNTPs, 0.8 μL of each primer (5 μM), 0.4 μL of FastPfu Polymerase, 0.2 μL of BSA and 10 ng of template DNA. The PCR products were extracted from a 2% agarose gel, purified using an AxyPrep DNA Gel Extraction Kit (Axygen Biosciences, Union City, CA, United States) and quantified using QuantiFluor^TM^-ST (Promega, Madison, WI, United States) according to the manufacturer’s instructions. Purified amplicons were pooled in equimolar concentrations and paired-end sequenced (2 × 300) on an Illumina MiSeq platform (Illumina, San Diego, CA, United States) according to the standard protocols by Majorbio Bio-Pharm Technology Co. Ltd. (Shanghai, China). The raw reads were deposited into the NCBI Sequence Read Archive (SRA) database (Accession Number: SRP148357).

### Processing of Sequencing Data

Raw FASTQ files were demultiplexed, quality-filtered by Trimmomatic and merged using FLASH with the following criteria: (1) the reads were truncated at any site receiving an average quality score < 20 over a 50 bp sliding window; (2) the primers were exactly matched allowing 2 nucleotide mismatching, and reads containing ambiguous bases were removed, and (3) sequences whose overlaps were longer than 10 bp were merged according to their overlap sequence. OTUs were clustered with a 97% similarity cutoff using UPARSE (version7.1)^1^ (Edgar, 2013), and chimeric sequences were identified and removed using UCHIME. The taxonomy of each ITS gene sequence was analyzed using the UNITE (version7.2)^2^ database with a confidence threshold of 70% (Koljalg et al., 2014).

The diversity indices Shannon and Chao1 were calculated and used to compare the fungal community alpha diversity. Principal coordinates analysis (PCoA) that utilized Bray_Curtis and Binary_Euclidean distance metrics was performed using OTUs for each sample. Linear discriminant analysis (LDA) effect size (LEfSe) was used to elucidate the biomarkers in each treatment. Those with an LDA score ≥ 2.0 were considered to be important biomarkers in each treatment. The cladogram was drawn using the Huttenhower Galaxy web application via the LEfSe algorithm^3^ (Segata et al., 2011).

### Statistical Analysis

Heat map analysis was used to reveal the significant differences of the dominant fungal genera among those with an average abundance greater than 0.5% in each treatment. The response ratio analysis was analyzed using STAMP^4^ to determine genera that had significant differences (Parks et al., 2014), and additionally to predict potentially pathogenic and beneficial fungi using FUNGuild^5^ (Nguyen et al., 2016). The pathogenic and beneficial fungi were assigned according to potential for damaging or benefiting the plant. Statistical analyses were performed using the multcomp package in R (3.4.1) (R Development Core Team, 2010). Pearson correlation analysis was used to test the correlation significance between fungal community and soybean yield, potentially pathogenic and beneficial fungi, soybean root disease incidence and the cysts of soybean cyst nematodes.

## Results

### Fungal Abundance and Community Composition

**FIGURE 1:**
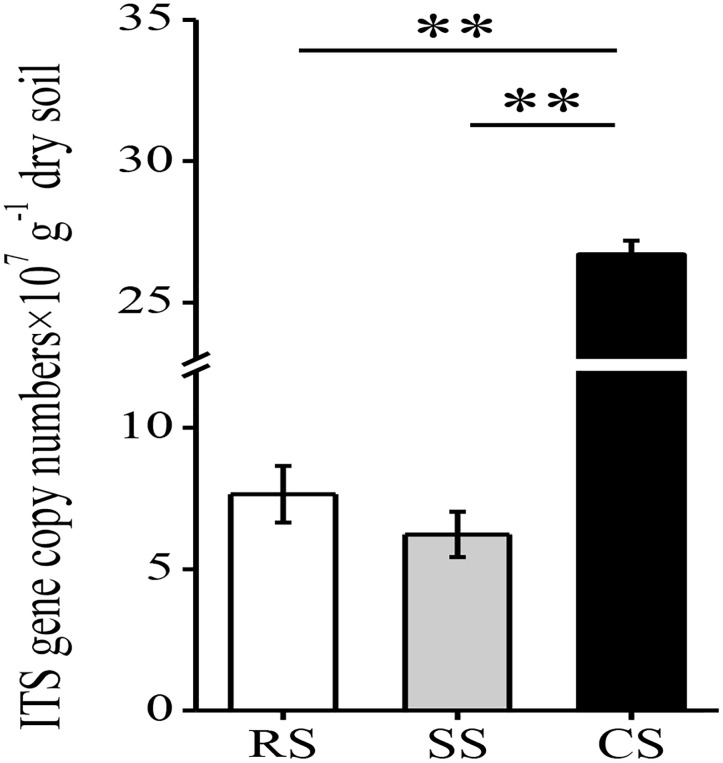
The abundance of fungal ITS rRNA gene copies assessed by real-time PCR in three soybean cropping systems. ^∗∗^*P* < 0.01 by Duncan’s test. RS, SS, and CS represented soybean rotation with a 3-year cycle of wheat, maize and soybean, short-term continuous soybean cropping with one-year wheat and two-year soybean, and long-term continuous soybean cropping, respectively.

The fungal abundance was quantified using the real-time PCR method. Compared with RS and SS, CS significantly increased the fungal abundance, which was 3.49- and 4.29-fold higher than that of RS and SS, respectively (Figure 1). The fungal abundance in SS was slightly lower than that of RS. In total, 328,986 quality sequences were obtained from all samples with 32,010–39,762 sequences per sample (mean = 36,554). The average lengths ranged from 211 to 435 bp with a mean of 259 bp (Supplementary Table S1). The OTUs identified in all samples were divided into 6 phyla, 20 classes, 55 orders, 106 families and 196 genera.

**FIGURE 2:**
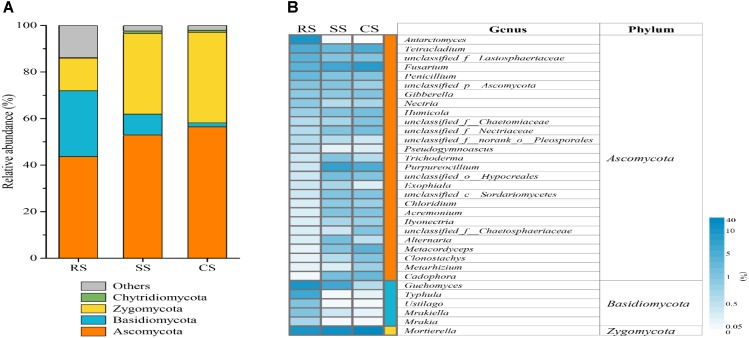
Relative abundance of different fungi at the phylum level **(A)** and heat map analysis **(B)** of the dominant fungal genera with relative abundance greater than 0.5% in three soybean cropping systems. The color code indicated relative abundance, ranging from white (low abundance) to blue (high abundance). RS, SS, and CS represented soybean rotation with a 3-year cycle of wheat, maize and soybean, short-term continuous soybean cropping with 1-year wheat and 2-year soybean, and long-term continuous soybean cropping, respectively.

There were different variations in fungal community compositions among RS, SS and CS. At the phylum level, the dominant fungal phyla in RS and SS were Ascomycota, Basidiomycota and Zygomycota, with relative abundances of 43.67 and 52.92%, 28.30 and 8.97%, 13.99 and 34.75% in RS and SS, respectively (Figure 2A and Supplementary Table S2). However, the dominant fungal phyla were Ascomycota and Zygomycota in CS with relative abundances of 56.42 and 38.92%, respectively. Chytridiomycota was a minor phylum with a lower relative abundance. Compared with RS, SS, and CS enhanced the relative abundances of Ascomycota by 21.18 and 29.21% and Zygomycota by 148.41 and 178.25%, respectively. The continuous soybean cropping SS and CS decreased the relative abundance of Basidiomycota by 68.32 and 93.84%, respectively.

At the genus level, there were a total of 33 genera with an average abundance greater than 0.5%, which accounted for 79.60–89.18% of the total population (Figure 2B). In RS, the three genera *Guehomyces* (16.86%), *Mortierella* (13.68%) and *Antarctomyces* (9.99%) were dominant, while in SS and CS, the three genera *Mortierella* (34.32, 38.56%), *Purpureocillium* (8.47, 5.72%) and *Fusarium* (7.47, 9.31%) were dominant, respectively (Supplementary Table S3). Compared to RS, CS and SS resulted in a significant increase in the relative abundance of *Purpureocillium, Ilyonectria, Clonostachys, Cadophora*, and *Mortierella*, while there was a significant decrease in the relative abundance of *Penicillium, Guehomyces, Ustilago, Mrakiella*, and *Mrakia*.

### Fungal Community Diversity

**FIGURE 3:**
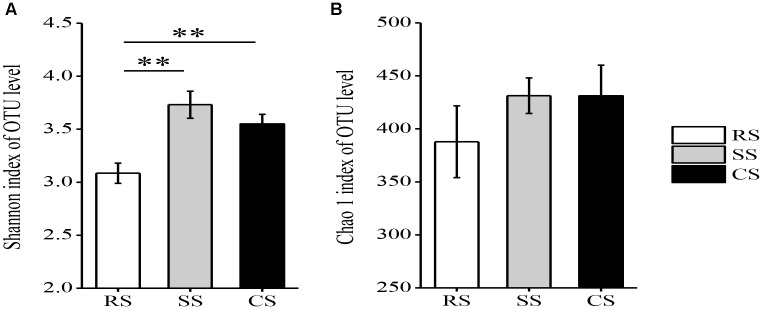
Shannon index **(A)** and Chao 1 index **(B)** for fungi in three soybean cropping systems with > 97% sequence identity. ^∗∗^*P* < 0.01 by Duncan’s test. RS, SS, and CS represented soybean rotation with a 3-year cycle of wheat, maize and soybean, short-term continuous soybean cropping with 1-year wheat and 2-year soybean, and long-term continuous soybean cropping, respectively.

The continuous cropping of SS and CS increased the fungal community diversity. The Shannon index was significantly higher in CS and SS than in RS (Figure 3A). The Chao1 index demonstrated that the upper limits of CS and SS were both larger than that of RS, but the differences were not statistically significant (Figure 3B).

**FIGURE 4:**
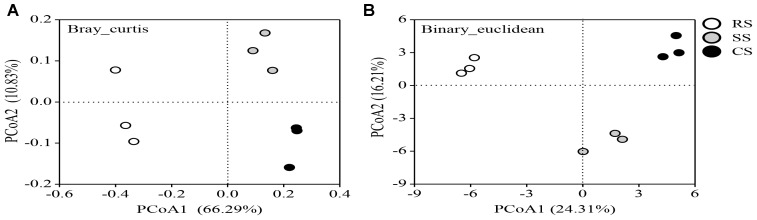
Principal coordinate analysis (PCoA) plot based on the OTUs of Bray **(A)** and Binary **(B)** for three soybean cropping systems. RS, SS, and CS represented soybean rotation with a 3-year cycle of wheat, maize and soybean, short-term continuous soybean cropping with 1-year wheat and 2-year soybean, and long-term continuous soybean cropping, respectively.

The PCoA plot based on the OTUs of the Bray and Binary analyses showed clear similarities or differences among the fungal community structures across all the samples (Figures 4A,B). The first two principal coordinates explained 77.12 and 40.52% of the total variance of the soil fungal community structures in Bray and Binary, respectively. All of the soil samples separated into two groups based on the PCoA analysis. CS and SS were grouped together and were clearly separated from RS, indicating that CS and SS had similar fungal community structures on PCoA1. In addition, SS and CS were distinctly separated from each other along the PCoA2.

**FIGURE 5:**
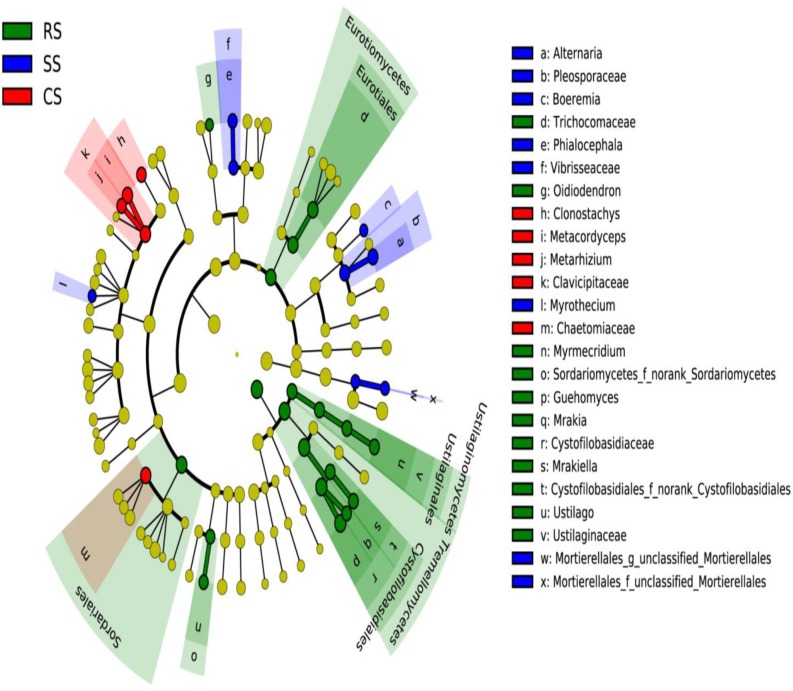
Taxonomic cladogram produced from the LEfSe analysis. The phylum, class, order, family, and genus levels are listed in order from inside to outside of the cladogram, and the labels for levels of the family and genus are abbreviated using a single letter. Red, green, and blue showed taxa enriched in CS, RS, and SS, respectively, while the yellow circles represented the taxa without significant differences among the three cropping systems. RS, SS, and CS represented soybean rotation with a 3-year cycle of wheat, maize and soybean, short-term continuous soybean cropping with 1-year wheat and 2-year soybean, and long-term continuous soybean cropping, respectively.

An analysis of the primary taxa, accounting for the differences of fungal composition across all the soil samples, was carried out using LEfSe analysis. The cladogram showed that a total of 32 characteristics had significantly different abundances among the three cropping systems at an LDA threshold of 2.0, including 4 classes, 4 orders, 10 families, and 14 genera. There were 5, 19, and 8 fungal taxa identified in CS, RS, and SS, respectively (Figure 5). At the genus level, RS was differentially enriched with the genera *Oidiodendron, Myrmecridium, Guehomyces, Mrakia, Mrakiella*, and *Ustilago*, and the LDA score of *Guehomyces* was higher than that of the other genera (Supplementary Figure S1). However, SS was enriched with *Alternaria, Boeremia, Phialocephala* and *Myrothecium*, among which *Alternaria* had by far the highest LDA score and played the most important role in SS. The continuous cropping CS was enriched with *Clonostachys, Metacordyceps*, and *Metarhizium*, and the LDA score of *Metacordyceps* was the highest among these three genera.

Soybean continuous cropping decreased soybean yield, and the soybean yield was significantly lower in CS than that in SS (Supplementary Figure S2). The relationship of fungal community with soybean yield was demonstrated by Pearson correlation (Table 1). The soybean yield was significantly negatively correlated with Shannon index and PCoA1 of the Bray and Binary analyses based on OTUs.

**Table 1:** Pearson correlation coefficients between fungal community diversity and soybean yield.

	Shannon	Chao	PC1 (Bray-Curtis)	PC2 (Bray-Curtis)	PC1 (Binary-Euclidean)	PC2 (Binary-Euclidean)
Soybean yield	-0.754^∗^	-0.394	-0.952^∗∗^	0.088	-0.953^∗∗^	0.016


*^∗^Indicates *P* < 0.05 level, ^∗∗^indicates *P* < 0.01 level.*

### Potentially Pathogenic and Beneficial Fungi

**FIGURE 6:**
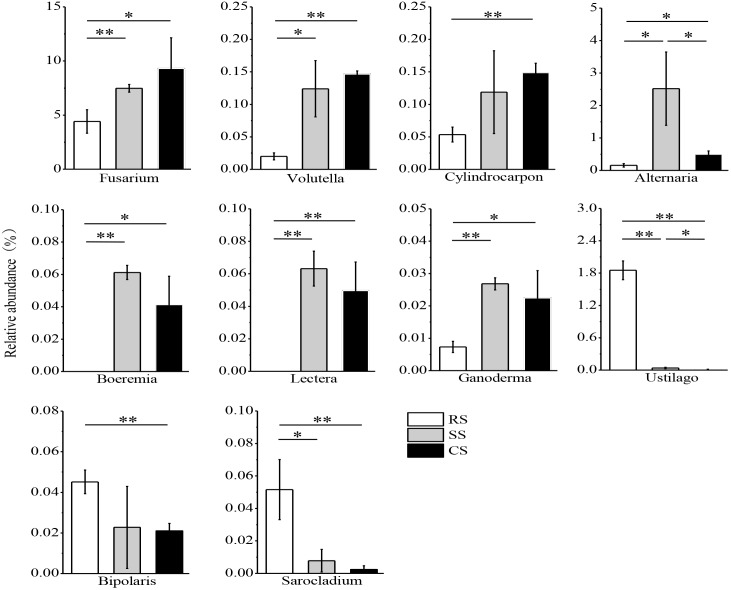
Relative abundance of potentially pathogenic fungi in three soybean cropping systems. ^∗^*P* < 0.05, ^∗∗^*P* < 0.01 by Student’s *t*-test (equal variance). RS, SS, and CS represented soybean rotation with a 3-year cycle of wheat, maize and soybean, short-term continuous soybean cropping with 1-year wheat and 2-year soybean, and long-term continuous soybean cropping, respectively.

**FIGURE 7:**
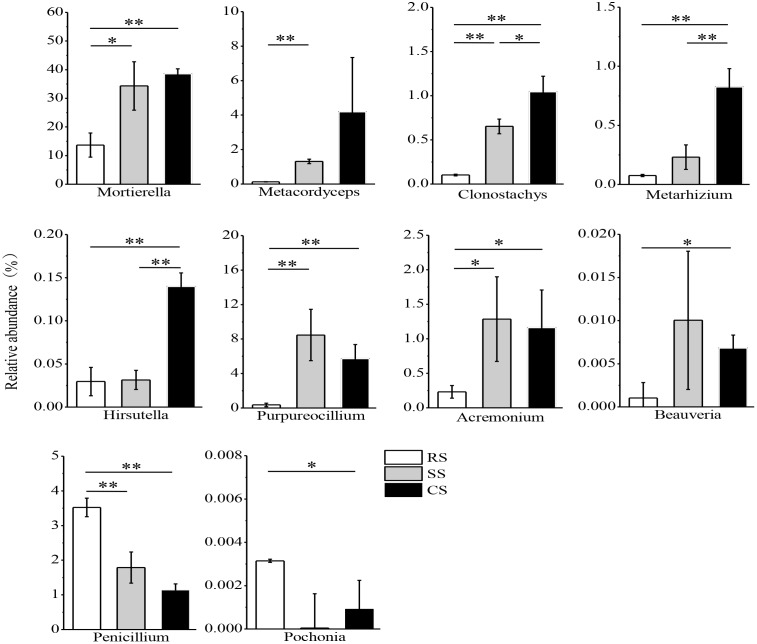
Relative abundance of potentially beneficial fungi in three soybean cropping systems. ^∗^*P* < 0.05, ^∗∗^*P* < 0.01 by Student’s *t*-test (equal variance). RS, SS, and CS represented soybean rotation with a 3-year cycle of wheat, maize and soybean, short-term continuous soybean cropping with 1-year wheat and 2-year soybean, and long-term continuous soybean cropping, respectively.

The variance of the fungal relative abundance between SS or CS and RS was illustrated by the response ratio analysis at the genus level (Supplementary Figures S3a,b). There were 41 genera significantly shifted in relative abundance in SS compared with RS with 29 genera increased and 12 genera decreased (Supplementary Figure S3a). The relative abundance of 55 genera varied in CS compared to RS with 38 genera increased and 17 genera decreased (Supplementary Figure S3b). Subsequent fungal guild analysis showed that 10 potentially pathogenic fungi and 10 potentially beneficial fungi shifted in SS and CS compared to RS (Supplementary Table S4). One of the most noteworthy findings in this study was that the relative abundance of the potentially pathogenic fungi *Fusarium, Volutella, Cylindrocarpon, Alternaria, Boeremia, Lectera*, and *Ganoderma* was dramatically enhanced in SS and CS compared to RS, while *Boeremia* and *Lectera* were not detected in RS (Figure 6). However, SS and CS caused a decrease in the potentially pathogenic fungi *Ustilago, Bipolaris* and *Sarocladium*. Compared to SS, CS decreased the relative abundance of both *Alternaria* and *Ustilago*. Among the potentially beneficial fungi, *Mortierella, Metacordyceps, Clonostachys, Metarhizium, Hirsutella, Purpureocillium, Acremonium*, and *Beauveria* had a higher relative abundance in SS and CS than in the RS (Figure 7). In contrast, the relative abundance of *Penicillium* and *Pochonia* decreased in SS and CS compared with that of RS. Between continuous soybean cropping systems, CS increased the relative abundance of *Clonostachys, Metarhizium*, and *Hirsutella* compared to SS.

Soybean continuous cropping influenced the soybean disease severities. SS increased soybean root disease incidence (DI) and the FC compared with RS, and both were higher in SS than in CS (Supplementary Figures S4, S5a). There was no difference in the DI and FC between CS and RS. Soybean continuous cropping increased the EC, and it was higher in CS than in SS (Supplementary Figure S5b). For potentially pathogenic fungi, the Pearson correlation analysis showed that the *Fusarium* positively correlated with the EC, and *Alternaria, Boeremia*, and *Lectera* were positively correlated with the DI and the FC (Table 2). For potentially beneficial fungi, *Mortierella, Metacordyceps, Clonostachys, Metarhizium*, and *Hirsutella* were positively but *Penicillium* was negatively correlated with the EC. Fungi *Purpureocillium* and *Beauveria* were positively correlated with the DI. The *Purpureocillium* was positively correlated with the FC.

**Table 2:** Pearson correlation coefficients between the relative abundance of potentially pathogenic and beneficial genera and soybean root disease incidence (DI), full cysts (FC), and empty cysts (EC) of soybean cyst nematodes.

	DI	FC	EC
**Potential pathogenic genera**			
*Fusarium*	0.336	0.160	0.695^*^
*Volutella*	0.524	0.357	0.627
*Cylindrocarpon*	0.572	0.288	0.650
*Alternaria*	0.712^*^	0.854^**^	-0.142
*Boeremia*	0.827^**^	0.737^*^	0.343
*Lectera*	0.843^**^	0.669^*^	0.403
*Ganoderma*	0.695^*^	0.661	0.468
*Ustilago*	-0.722^*^	-0.552	-0.627
*Bipolaris*	-0.375	-0.304	-0.421
*Sarocladium*	-0.616	-0.447	-0.612
**Potential beneficial genera**			
*Mortierella*	0.663	0.451	0.686^*^
*Metacordyceps*	-0.019	-0.062	0.938^**^
*Clonostachys*	0.483	0.160	0.721^*^
*Metarhizium*	0.126	-0.232	0.794^*^
*Hirsutella*	-0.091	-0.399	0.838^**^
*Purpureocillium*	0.736^*^	0.684^*^	0.319
*Acremonium*	0.615	0.413	0.220
*Beauveria*	0.777^*^	0.599	0.228
*Penicillium*	-0.546	-0.348	-0.779^*^
*Pochonia*	-0.413	-0.323	-0.417


*^∗^Indicates *P* < 0.05 level, ^∗∗^indicates *P* < 0.01 level.*

## Discussion

### Shifts in Fungal Abundance and Community Composition in Response to Long-Term Continuous Soybean Cropping

In this study, CS led to an increase in soil fungal abundance compared with the RS system and SS (Figure 1). This is consistent with another study which showed that the cultivable fungal population in the continuous soybean cropping was higher than that in the RS and that the imbalance of soil microbial flora occurred in the continuous cropping soil (Meriles et al., 2009). Zhou and Wu (2012) used q-PCR analysis and found that continuous cucumber cropping increased the fungal abundance in the rhizosphere. The increased fungal abundance may be related to the root exudates and different response of the microbes to root exudates or rhizodepositions. In this study, soybean roots exuded a number of flavonoids and phenolic acids (Colpas et al., 2003; Guo et al., 2011), which were lower in the wheat and maize root exudates of the RS or SS. It was found that flavonoids and phenolic acids had a significant effect on the fungal community, especially fungal pathogens (Qu and Wang, 2008; Wang et al., 2013). Overall, our study suggested that CS promotes soil fungal growth.

The effects of CS on the soil fungal community composition were distinctly observed at the phylum and genus level. At the phylum level, Ascomycota was the most abundant, and its relative abundance increased in SS and CS compared to RS (Figure 2A), suggesting the ubiquity and important role of Ascomycota in the soybean ecosystems. This is consistent with a previous study that found that Ascomycota was the most important phylum in continuous soybean cropping soils (Li et al., 2010). Both SS and CS enhanced the relative abundance of Zygomycota (Figure 2A), which is a saprophytic phylum and plays an important role in decomposing plant debris (Richardson, 2009). This agrees with the findings of (Li Z. et al., 2016), who found that the relative abundance of Ascomycota, Zygomycota, and Glomeromycota increased with continuous *Piper nigrum* L. (black pepper) cropping duration. At the genus level, our results demonstrated that CS and SS enhanced the relative abundance of some genera, such as *Fusarium, Humicola, Alternaria, Clonostachys*, and *Mortierell*a (Figure 2B). A previous study also reported that short-term continuous soybean cropping caused an increase in the relative abundance of *Fusarium, Humicola*, and *Alternaria* (Bai et al., 2015). Continuous cropping of other plants, such as *Pseudostellaria heterophylla*, also increased the relative abundance of *Fusarium, Clonostachys* and *Mortierella* (Wu et al., 2016). Our results indicated that CS affected the fungal composition and altered the dominant genera. The causes of variation in the microbial community composition are multifaceted. For example, they can include soil nutrient imbalances, soil physical and chemical property deterioration and allelopathic autotoxicity accumulation (Kong et al., 2008; Li et al., 2010). However, many studies indicated that the changes of the microbial community composition are caused by the indirect ecological effect of root exudates rather than direct allelopathic autotoxicity (Guo et al., 2011; Wu et al., 2015). Owing to the different components of root exudates, some of them may promote the reproduction of certain microorganisms, such as soil-borne pathogens, while others may benefit the reproduction of beneficial microbes, such as antagonistic microorganisms in this manner, resulting in an increase of corresponding chemotaxis microbes (Li et al., 2014a).

### Fungal Diversity in Response to Long-Term Continuous Soybean Cropping

An increase in the Shannon index was observed in SS and CS, suggesting that continuous soybean cropping increased the soil fungal diversity (Figure 3A). Bai et al. (2015) and Xiong et al. (2015) also reported that the continuous cropping of soybean and vanilla (*Vanilla planifolia*) dramatically enhanced the fungal diversity. However, there are also contrasting findings that the fungal community diversity was not different between the continuous cropping of soybean/potato (*Solanum tuberosum* L.) and their corresponding rotation systems (Li et al., 2010; Liu et al., 2014). This might due to inconsistent technology or crop and soil environments, since different crops have diverse root exudates that affected the microbial community (Rengel, 1997).

In this study, Bray_Curtis and Binary_Euclidean were used to evaluate the beta-diversity of the fungal community. The Bray_Curtis considered both species composition and the fungal abundance, while the Binary_Euclidean only considered fungal species composition. The Bray and Binary principal coordinate analyses indicated that continuous soybean cropping resulted in the greatest impact on the variation of the fungal community structure. Along the PCoA1, both Bray_Curtis and Binary_Euclidean showed a clear separation between the continuous soybean cropping and the RS system, indicating that continuous soybean cropping changed the fungal community structure both in species composition and the abundance of fungi (Figures 4A,B). However, 2-year and long-term continuous soybean cropping were separated on the PCoA2 axis, suggesting that the soil fungal communities are influenced by the duration of continuous cropping. This finding is generally consistent with the recent investigation in continuous peanut cropping systems, which demonstrated that the fungal community composition and structure of 5-year and 10-year continuous peanut were similar compared to 1 year of continuous peanut cropping, but 5-year and 10-year continuous peanut cropping were also separated on the PC2 axis of the principal component analysis (Li et al., 2014b). This is likely due to the differential accumulation in the amount of crop root exudates and residues in the rhizosphere microenvironment with continuous cropping duration, since crop root exudates and residues can influence the microbial community structure by serving as substances for microbes. Subsequent LEfSe analysis showed that the change in the fungal community structure was primarily driven by 14 genera, among which *Guehomyces, Alternaria*, and *Metacordyceps* played an important role in RS, SS, and CS, respectively (Supplementary Figure S1). The Pearson correlation analysis between the soybean yield and fungal community diversity showed a negative relationship between the soybean yield and the Shannon index and the PCoA1 of the Bray and Binary analyses (Table 1). The Bray_Curtis considered both species composition and the fungal abundance. Thus, this result suggested that the soybean productivity is negatively correlated with the fungal species composition and abundance.

### Changes in Potentially Pathogenic and Beneficial Fungi in Response to Long-Term Continuous Soybean Cropping

This study found that the relative abundance of potentially pathogenic fungi significantly increased in SS and CS, indicating continuous soybean cropping benefits the proliferation of specific pathogenic fungi. Our finding demonstrated that SS and CS significantly increased the relative abundance of *Fusarium* and *Alternaria* (Figure 6). *Fusarium* is well known to be an pathogenic fungus that leads to soybean *Fusarium* root rot (Zhang et al., 2010; Chang et al., 2018), and *Alternaria* can infect various crops and cause corresponding diseases, such as soybean Alternaria leaf spot, tomato and carrot black rots, citrus fruit gray rot and cereal black point (Logrieco et al., 2009; Li and Yang, 2009). In our study, *Alternaria* also showed a positive correlation with the soybean root disease incidence. This finding suggested that continuous soybean cropping could exacerbate crop disease. This is consistent with a previous study that the dominant pathogenic fungi were *Fusarium* and *Alternaria* in soybean fields, and their abundance was higher in 1-year and 2-year continuous soybean cropping than in RS soil (Bai et al., 2015). *Fusarium* showed a significantly positive correlation with the ECs but non-significant correlation with soybean root disease incidence (Table 2). This may be due to the complexity of soybean root rot, caused by a variety of pathogens; *Fusarium* is a pathogen but can also infect the cysts or eggs of soybean cyst nematodes (Song et al., 2017). In this study, the relative abundance of *Volutella, Cylindrocarpon*, and *Ganoderma* was more enriched in SS and CS and much less abundant in the RS (Figure 6), especially *Ganoderma* was significantly positively correlated with the soybean root disease incidence (Table 2). These three fungi were designated to potentially pathogenic fungi based on FUNGuild and relative literature^6^ (Gilbertson and Ryvarden, 1987; Tedersoo et al., 2014). Some fungi of *Volutella*, such as *V. colletotrichoides*, were described in diseased alfalfa (*Medicago sativa* L.) and other forage legumes in Iowa (Cannon et al., 2012). Species of the fungus *Cylindrocarpon*, such as *C*. *destructans* var. *destructans*, can cause black root rot of Sanqi (*Panax notoginseng*) and grapevine (*Vitis* sp.) (Alaniz et al., 2007; Mao et al., 2014). *Ganoderma*, such as *G*. *boninense*, is a pathogen of oil palm (*Elaeis guineensis*) (Najihah et al., 2015). Both *Boeremia* and *Lectera* were not detected in RS, while their relative abundances were dramatically increased in SS and CS (Figure 6), and they were positively correlated with the soybean root disease incidence (Table 2). Some studies have reported that *Boeremia* caused stem rot of *Origanum dubium* in Oregano (*Origanum dubium* Boiss) and black rot of artichoke (*Cynara scolymus*) in California (Koike et al., 2016; Samouel et al., 2016). *Lectera*, a new genus of *Plectosphaerellaceae*, is a legume pathogen (Cannon et al., 2012). The relative abundance of most of the potentially pathogenic fungi increased in SS and CS compared to RS. However, there were relative abundances of some potentially pathogenic fungi, such as *Ustilago, Bipolaris* and *Sarocladium*, which decreased in SS and CS (Figure 6). This is likely due to the tendency of *Ustilago, Bipolaris* and *Sarocladium* to infect graminaceous crops. Therefore, their relative abundances decreased during continuous soybean cropping but increased in a RS system of wheat/maize/ soybean. Many studies have reported that these three fungi cause diseases of graminaceous crops, such as rice sheath rot caused by *Sarocladium oryzae* (Saravanakumar et al., 2009), maize smut disease caused by *Ustilago maydis* and maize leaf spot caused by *Bipolaris* (Kämper et al., 2006; Li G.F. et al., 2016). The above findings can also explain the negative correlation between *Ustilago* and the soybean root disease incidence (Table 2). Our finding also suggested that continuous soybean cropping duration results in selective pressure on some pathogenic fungi of crop.

Biocontrol microbes have been proposed to be a sustainable alternative to chemical control (Lecomte et al., 2016). In this study, the relative abundance of the most potentially beneficial fungi exhibited an increased trend in SS and CS (Figure 7). These beneficial fungi included fungi antagonistic to plant pathogen and parasitic fungi of insect and plant parasitic nematodes. Some species of *Mortierella* and *Clonostachys* are fungal antagonists to plant pathogens, and they can protect banana (*Musa* sp.) and soybean from *Fusarium* wilt disease and *Fusarium* root rot (Pan et al., 2013; Shen et al., 2018). Hamid et al. (2017) also found that *Clonostachys* was abundant during continuous soybean cropping and gradually increased with the duration of continuous cropping. Some species of *Metacordyceps, Metarhizium*, and *Beauveria* genera are parasitic fungi of insects, and they also have potential value as pharmaceuticals (Roberts and St. Leger, 2004; Kepler et al., 2012). A recent study demonstrated that *Beauveria bassiana* had negative effects on cotton (*Gossypium hirsutum*) aphid reproduction (Lopez et al., 2014). The fungi *Hirsutella, Purpureocillium* and *Pochonia* are parasitic fungi of plant parasitic nematodes. A previous study reported that *Hirsutella* combined with chitosan suppressed the infestation of soybean cyst nematodes in soybean roots (Mwaheb et al., 2017). *Purpureocillium* is not only a well-known beneficial agent against various plant pathogenic in agriculture but also a commercialized agent to control plant parasitic nematodes (Wang et al., 2016). Hamid et al. (2017) reported that the relative abundance of *Purpureocillium* gradually increased with the duration of soybean monoculture, and they also detected *Hirsutella* in CS soils. In this study, the relative abundance of *Pochonia* decreased in SS and CS compared with the RS (Figure 7). Song et al. (2016) found that the percentage of *Pochonia chlamydosporia* was significantly higher in CS system than in a RS system. These inconsistent results might be due to different experimental methods and the isolated positions of the fungi. Song et al. (2016) used the traditional method to isolate fungi from the cysts of the soybean cyst nematodes, while we used the high-throughput sequencing technique to analyze the relative abundance and composition of the fungi from the soybean field soils. Current results revealed that the relative abundance of *Penicillium* gradually decreased in RS, SS, and CS (Figure 7). This is consistent with the previous study of Wu et al. (2016) who found that continuous *Pseudostellaria heterophylla* cropping markedly reduced the relative abundance of *Penicillium*. Although some *Penicillium* species can cause crop diseases, many species of *Penicillium* have been found to be beneficial fungi, which can inhibit the growth of pathogenic organisms to control plant diseases (Van Wees et al., 2008). In this study, the relative abundance of *Clonostachys, Metarhizium* and *Hirsutella* significantly increased in CS compared to SS (Figure 7), which indicated that these potentially beneficial fungi were sensitive to continuous soybean cropping duration.

The Pearson correlation analysis demonstrated that *Purpureocillium* had positive correlation with the soybean root disease severity and the FC (Table 2), which suggested that the potentially beneficial fungi will increase accompanying the increase of pathogenic agents. The *Mortierella, Metacordyceps, Clonostachys, Metarhizium*, and *Hirsutella* were positively correlated with the ECs (Table 2). This is likely due to the parasitism of these potential beneficial fungi on the cysts or eggs of the soybean cyst nematodes (Song et al., 2017). This result is in agreement with previous study that *Hirsutella* can suppress soybean cyst nematodes (Mwaheb et al., 2017). Overall, these findings suggested that CS can increase the both pathogenic and beneficial fungi of plant and lead to an antagonistic development of the fungal community structure on plant health.

## Conclusion

In this study, CS increased soil fungal abundance compared with the RS system and SS. The Shannon index indicated that both long- and short-term continuous soybean cropping could increase soil fungal diversity. Continuous soybean cropping influenced the fungal community structure in species composition and the abundance of fungi with a clear separation between continuous soybean cropping and the RS system on the PCoA1 axis. The separation between 2-year and long-term continuous soybean cropping on the PCoA2 axis indicated that the continuous cropping duration also affected the soil fungal community. The change in the fungal community structure was primarily driven by 14 genera, and *Guehomyces, Alternaria*, and *Metacordyceps* contributed more to the variation in the RS and 2-year and long-term continuous soybean cropping, respectively. The relative abundance of most of the potentially pathogenic and beneficial fungi, including potential fungi antagonistic to plant pathogens and the parasitic fungi of insect and plant parasitic nematodes, increased during 2-year and long-term continuous soybean cropping. Overall, this study suggested that CS increased the fungal abundance and diversity and changed the fungal community composition compared to the RS. In addition, CS can result in the development of a fungal community structure that is antagonistic to plant health.

## Author Contributions

XH and FS designed the study. HL and FP performed the work. ZZ and JY analyzed the data. YX revised the manuscript. All authors read and approved the final manuscript.

## Conflict of Interest Statement

The authors declare that the research was conducted in the absence of any commercial or financial relationships that could be construed as a potential conflict of interest.

## Footnotes

1 http://drive5.com/uparse/

2 https://unite.ut.ee/

3 http://huttenhower.sph.harvard.edu/lefse/

4 http://kiwi.cs.dal.ca/Software/STAMP

5 http://www.stbates.org/guilds/app.php

6 https://github.com/UMNFuN/FUNGuild

## Abbreviations

CS: long-term continuous soybean cropping
DI: soybean root disease incidence
EC: empty cysts of soybean cyst nematodes
FC: full cysts of soybean cyst nematodes
OTUs: operational taxonomic units
PCoA: principal coordinate analysis
Q-PCR: quantitative real-time PCR
RS: soybean rotation
SS: 2-year continuous soybean cropping
