# Supplementary figures and images for: Response of Soil Fungal Community Structure to Long-Term Continuous Soybean Cropping
Source: Front Microbiol. 2019 Jan 9;9:3316. doi: 10.3389/fmicb.2018.03316 (PMC6333693; doi:10.3389/fmicb.2018.03316)

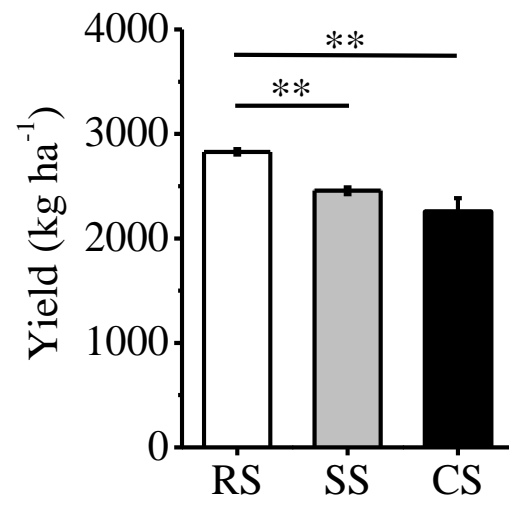

**FIGURE S2** | Soybean yield in three soybean cropping systems. \*\*P<0.01 by Duncan's test.

Supplement: Supplementary file 1 [file Data_Sheet_1.PDF]

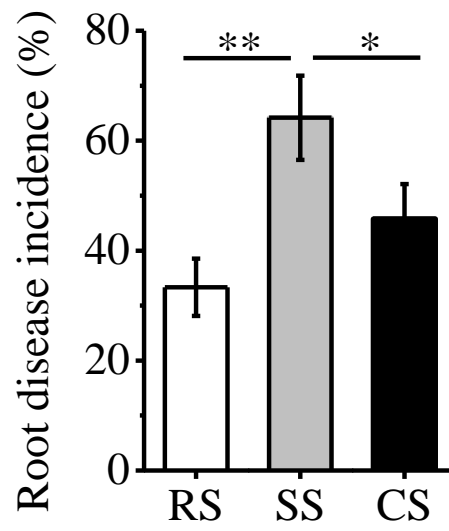

**FIGURE S4** | Soybean root disease incidence in three soybean cropping systems. \* $P < 0.05$ , \*\* $P < 0.01$  by Duncan's test.

Supplement: Supplementary file 2 [file Data_Sheet_4.PDF]
