# Supplementary material for: Response of Soil Fungal Community Structure to Long-Term Continuous Soybean Cropping
Source: Front Microbiol. 2019 Jan 9;9:3316. doi: 10.3389/fmicb.2018.03316 (PMC6333693; doi:10.3389/fmicb.2018.03316)
Supplement: Supplementary file 3 [file Data_Sheet_5.PDF]

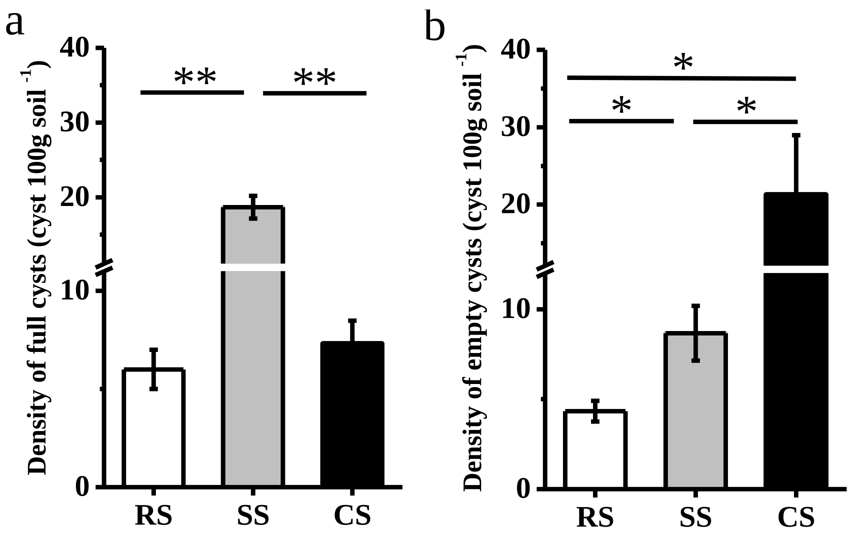

FIGURE S5 | Density of full cysts (a) and empty cysts (b) of soybean cyst nematodes in three soybean cropping systems. *P<0.05, **P<0.01 by Duncan's test.
