# Supplementary material for: Response of Soil Fungal Community Structure to Long-Term Continuous Soybean Cropping
Source: Front Microbiol. 2019 Jan 9;9:3316. doi: 10.3389/fmicb.2018.03316 (PMC6333693; doi:10.3389/fmicb.2018.03316)
Supplement: Supplementary file 4 [file Data_Sheet_6.PDF]

TABLE S1 | Illumina MiSeq sequenced fungal data (at 97% sequence similarity) based on the ITS rRNA gene in the three soybean cropping systems.

	Sequences	Bases(bp)	Average Length	Minimum length	Maximun length
RS	32010	8481468	265	211	412
SS	37889	9701521	256	215	435
CS	39762	10165547	256	216	435
