# Supplementary material for: Response of Soil Fungal Community Structure to Long-Term Continuous Soybean Cropping
Source: Front Microbiol. 2019 Jan 9;9:3316. doi: 10.3389/fmicb.2018.03316 (PMC6333693; doi:10.3389/fmicb.2018.03316)
Supplement: Supplementary file 5 [file Data_Sheet_7.PDF]

TABLE S2 | Relative abundance (%) of fungal phyla in three soybean cropping systems.

Phylum	RS	SS	CS
Ascomycota	43.67 ±14.76	52.92 ±9.06	56.42 ±4.17
Basidiomycota	28.3 ±13.39a	8.97 ±2.06b	1.74 ±1.30b
Zygomycota	13.99 ±4.42b	34.75 ±8.44a	38.92 ±1.76a
Chytridiomycota	0.23 ±0.18	1.02 ±0.93	0.9 ±1.34
Others	13.81 ±9.89a	2.35 ±0.73b	2.01 ±0.30b

Means ± standard deviation with different letters indicate significant differences using ANOVA($p < 0.05$)
