# Supplementary material for: Response of Soil Fungal Community Structure to Long-Term Continuous Soybean Cropping
Source: Front Microbiol. 2019 Jan 9;9:3316. doi: 10.3389/fmicb.2018.03316 (PMC6333693; doi:10.3389/fmicb.2018.03316)
Supplement: Supplementary file 6 [file Data_Sheet_8.PDF]

TABLE S3 | Relative abundance (%) of fungal genera in three soybean cropping systems with relative abundance greater than 0.5%.

**Phylum**	**Genus**	**RS**	**SS**	**CS**
Ascomycota	*Antarctomyces*	9.99±17.16	0.00±0.00	0±0
	Tetracladium	5.28±1.71	3.86±1.49	5.13±1.14
	unclassified_f__Lasiosphaeriaceae	4.87±1.47a	1.17±0.51c	1.41±0.76b
	Fusarium	4.41±1.08b	7.47±0.36ab	9.31±2.84a
	Penicillium	3.52±0.27a	1.79±0.45bc	1.14±0.18c
	unclassified_p__Ascomycota	1.07±0.07ab	1.42±0.35a	0.8±0.16b
	Gibberella	0.91±0.11b	0.92±0.12b	1.43±0.27a
	Nectria	0.87±0.20	0.59±0.20	0.69±0.14
	Humicola	0.76±0.29b	1.45±0.44ab	2.19±0.46a
	unclassified_f__Chaetomiaceae	0.62±0.18b	0.7±0.10ab	1.06±0.15a
	unclassified_f__Nectriaceae	0.62±0.08c	1.8±0.19ab	2.33±0.64a
	unclassified_f__norank_o__Pleosporales	0.61±0.33	0.64±0.15	0.37±0.06
	Pseudogymnoascus	0.52±0.22	0.08±0.08	0.18±0.25
	Trichoderma	0.41±0.06	1.45±0.77	0.6±0.29
	Purpureocillium	0.36±0.20c	8.47±2.99a	5.72±1.64ab
	unclassified_o__Hypocreales	0.35±0.04b	2.15±0.41a	1.23±0.74ba
	Exophiala	0.34±0.05b	0.67±0.20a	0.21±0.07b
	unclassified_c__Sordariomycetes	0.31±0.16	1.35±0.54	1.77±2.74
	Chloridium	0.25±0.10b	0.9±0.30a	0.77±0.27ab
	Acremonium	0.23±0.09	1.29±0.61	1.16±0.55
	Ilyonectria	0.22±0.03c	0.68±0.21ab	0.79±0.10a
	unclassified_f__Chaetosphaeriaceae	0.16±0.09b	0.38±0.11ab	1.2±0.60a
	Alternaria	0.16±0.05b	2.52±1.13a	0.49±0.11b
	Metacordyceps	0.13±0.00	1.31±0.13	4.19±3.15
	Clonostachys	0.1±0.01c	0.65±0.08b	1.04±0.18a
	Metarhizium	0.08±0.01c	0.23±0.10bc	0.83±0.15a
	Cadophora	0.01±0.01c	2.29±0.74ab	3.89±1.20a
Basidiomycota	*Guehomyces*	16.86±1.04a	7.18±1.53b	0.56±0.22c
	Typhula	7.01±12.11	0.04±0.03	0.05±0.05
	Ustilago	1.85±0.17a	0.04±0.01b	0.01±0.00b
	Mrakiella	1.32±0.15a	0.31±0.07b	0.07±0.03c
	Mrakia	0.72±0.18a	0.05±0.01b	0.00±0.00b
Zygomycota	*Mortierella*	13.68±4.23a	34.32±8.44b	38.56±1.73b

Means ± standard deviation with different letters indicate significant differences using an ANOVA($p < 0.05$)
