# Supplementary material for: Response of Soil Fungal Community Structure to Long-Term Continuous Soybean Cropping
Source: Front Microbiol. 2019 Jan 9;9:3316. doi: 10.3389/fmicb.2018.03316 (PMC6333693; doi:10.3389/fmicb.2018.03316)
Supplement: Supplementary file 7 [file Data_Sheet_9.pdf]

TABLE S4 | The guild of potentially pathogenic fungi and potentially beneficial fungi.

**Potentially pathogenic fungi**	**Guild**	**Reference**
Fusarium	Root rot of soybean	Zhang JX, Xue AG, Zhang HJ, Nagasawa AE, Tambong JT, 2010. Response of soybean cultivars to root rot caused by *Fusarium* species. Canadian Journal of Plant Science 90,767-776.
Volutella	Legume pathogen	Cannon P, Buddie A, Bridge P, Neergaard ED, 2012. Lectera, a new genus of the Plectosphaerellaceae for the legume pathogen Volutella colletotrichoides. Mycokeys 3, 23-36.
Cylindrocarpon	Black root rot of Sanqi	Mao ZS, Long YJ, Zhu YY, Zhu SS, He XH, 2014. First report of *Cylindrocarpon destructans* var. *destructans* causing black root rot of Sanqi (*Panax notoginseng*) in China. Plant Disease 98, 162-162.
	Black Foot Disease of Grapevine	Alaniz S, León M, Vicent A, García-Jiménez J, Abadcampos P, Armengol J, 2007. Characterization of *Cylindrocarpon* Species Associated with Black Foot Disease of Grapevine in Spain. Plant Disease 91, 1187-1193.
Alternaria	Alternaria leaf spot of soybean	Li X, Yang XB, 2009. Similarity, pattern, and grouping of soybean fungal diseases in the United States: implications for the risk of soybean rust. Plant Disease 93, 162-169.
Sarocladium	Rice sheath rot disease	Saravanakumar D, Raguchander T, Samiyappan R, 2009. Fluorescent pseudomonad mixtures mediate disease resistance in rice plants against sheath rot (*Sarocladium oryzae*) disease.Biocontrol 54, 273-286.
Boeremia	Black rot of artichoke	Koike ST, Groenewald JZ, Crous PW, 2016. First report of black rot caused by *Boeremia exigua* var. *pseudolilacis* on artichoke in California. Plant Disease 100, 524.
	Stem rot of Origanum dubium	Samouel S, Iacovides T, Evangelides S, Kanetis L, 2016. First report of *Boeremia exigua* var. *exigua* causing stem rot of Origanum dubium in Cyprus. Plant Disease 100, 529.
Lectera	Legume pathogen	Cannon P, Buddie A, Bridge P, Neergaard ED, 2012. *Lectera*, a new genus of the *Plectosphaerellaceae* for the legume pathogen *Volutella colletotrichoides*. Mycokeys 3, 23-36.
Ganoderma	Basal stem rot disease of oil palm	Najihah NI, Hanafi MM, Idris AS, Hakim MA, 2015. Silicon treatment in oil palms confers resistance to basal stem rot disease caused by *Ganoderma boninense*. Crop Protection 67, 151-159.
Ustilago	Common smut of maize	Kamper J, Kahmann R, Bolker M, Ma LJ, Brefort T, Saville B J, et al., 2006. Insights from the genome of the biotrophic fungal plant pathogen Ustilago maydis. Nature 444, 97-101.
Bipolaris	Leaf spot of maize	Li GF, Liu KX, Xiao Q, Lu YY, Xue CS, Wang GQ. 2016a. First report of leaf spot of maize (*Zea mays*) caused by *Bipolaris spicifera* in China. Plant Disease 100, 855.
**Potentially beneficial fungi**	**Guild**	**Reference**

Mortierella	Insect pathogens	Edgington S, Thompson E, Moore D, Hughes KA, Bridge P, 2014. Investigating the insecticidal potential of Geomyces (*Myxotrichaceae* : *helotiales*) and mortierella (*Mortierellacea* : *mortierellales*) isolated from Antarctica. Springerplus 3, 1-8.
Metacordyceps	Insect pathogens	Kepler RM, Sung GH, Ban S, Nakagiri A, Chen MJ, Huang B, Li Z, Spatafora JW, 2012. New teleomorph combinations in the entomopathogenic genus *Metacordyceps* . Mycologia 104, 182-197.
Clonostachys	Nematode pathogens	Pan F, Xue AG, Mclaughlin NB, Li S, Xu Y, Zhao D, Qu H, 2013. Colonization of *Clonostachys rosea* on soybean root grown in media inoculated with *Fusarium graminearum* . Acta Agriculturae Scandinavica 63, 564-569.
Metarhizium	Insect pathogens	Roberts DW, Leger RJS, 2004. *Metarhizium* spp., Cosmopolitan Insect-Pathogenic Fungi: Mycological Aspects. Advances in Applied Microbiology 54, 1.
Hirsutella	Nematode pathogens	Mai AMAM, Hussain M, Tian J, Zhang X, Hamid MI, El-Kassim NA, Hassan GM, Xiang M, Liu XZ, 2017. Synergetic suppression of soybean cyst nematodes by chitosan and *Hirsutella minnesotensis* via the assembly of the soybean rhizosphere microbial communities. Biological Control 115, 85-94.
Purpureocillium	Nematode pathogens	Wang G, Liu Z, Lin R, Li E, Mao Z, Ling J, Yang Y, Yin W, Xie B, 2016. Biosynthesis of Antibiotic Leucinostatins in Bio-control Fungus *Purpureocillium lilacinum* and Their Inhibition on Phytophthora Revealed by Genome Mining. Plos Pathogens 12, e1005685.
Acremonium	Nematode pathogens	Singh S, Mathur N, 2010. Biological control of root-knot nematode, Meloidogyne incognita infesting tomato. Biocontrol Science and Technology 20, 865-874.
Beauveria	Insect pathogens	Castillo DL, Zhusalzman K, Ekramos MJ, Sword GA, 2014. The Entomopathogenic Fungal Endophytes *Purpureocillium lilacinum* (Formerly *Paecilomyces lilacinus*) and *Beauveria bassiana* negatively affect cotton aphid reproduction under both greenhouse and field Conditions. Plos One 9, e103891.
Penicillium	Nematode pathogens	Wees SC, Ent SV. Pieterse CM, 2008. Plant immune responses triggered by beneficial microbes. Current Opinion in Plant Biology 11, 443-448.
Pochonia	Nematode pathogens	Hamid MI, Hussain M, Wu Y, Zhang X, Xiang M, Liu X, 2017. Successive soybean-monoculture cropping assembles rhizosphere microbial communities for the soil suppression of soybean cyst nematode. FEMS Microbiology Ecology 93, fiw222.
