# Supplementary material for: Response of Soil Fungal Community Structure to Long-Term Continuous Soybean Cropping
Source: Front Microbiol. 2019 Jan 9;9:3316. doi: 10.3389/fmicb.2018.03316 (PMC6333693; doi:10.3389/fmicb.2018.03316)
Supplement: Supplementary file 8 [file Image_1.pdf]

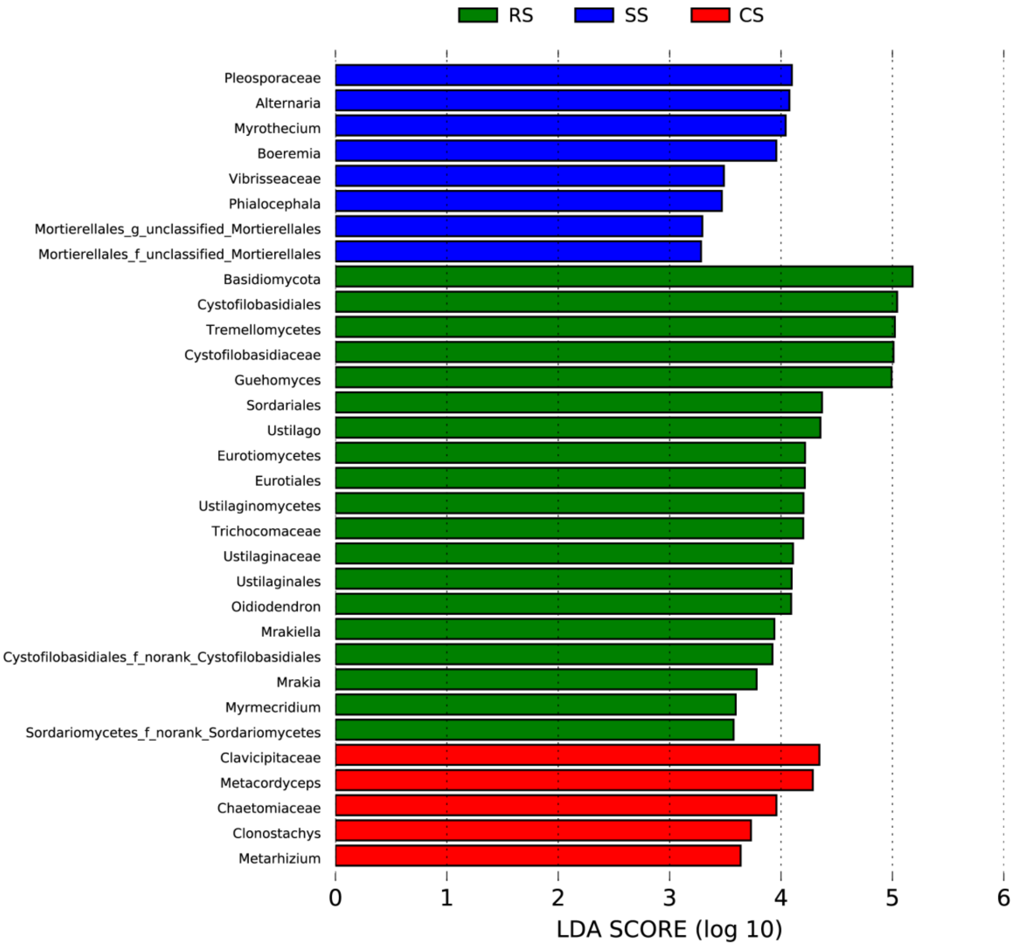

Figure S1 | Histogram of the linear discriminant analysis (LDA) scores. Taxa enriched in CS are shown in red with a positive LDA score, RS in green with positive LDA score and SS in blue with a positive LDA score ($P < 0.05$; LDA score 2.0).
