# Supplementary material for: Response of Soil Fungal Community Structure to Long-Term Continuous Soybean Cropping
Source: Front Microbiol. 2019 Jan 9;9:3316. doi: 10.3389/fmicb.2018.03316 (PMC6333693; doi:10.3389/fmicb.2018.03316)
Supplement: Supplementary file 9 [file Image_3.pdf]

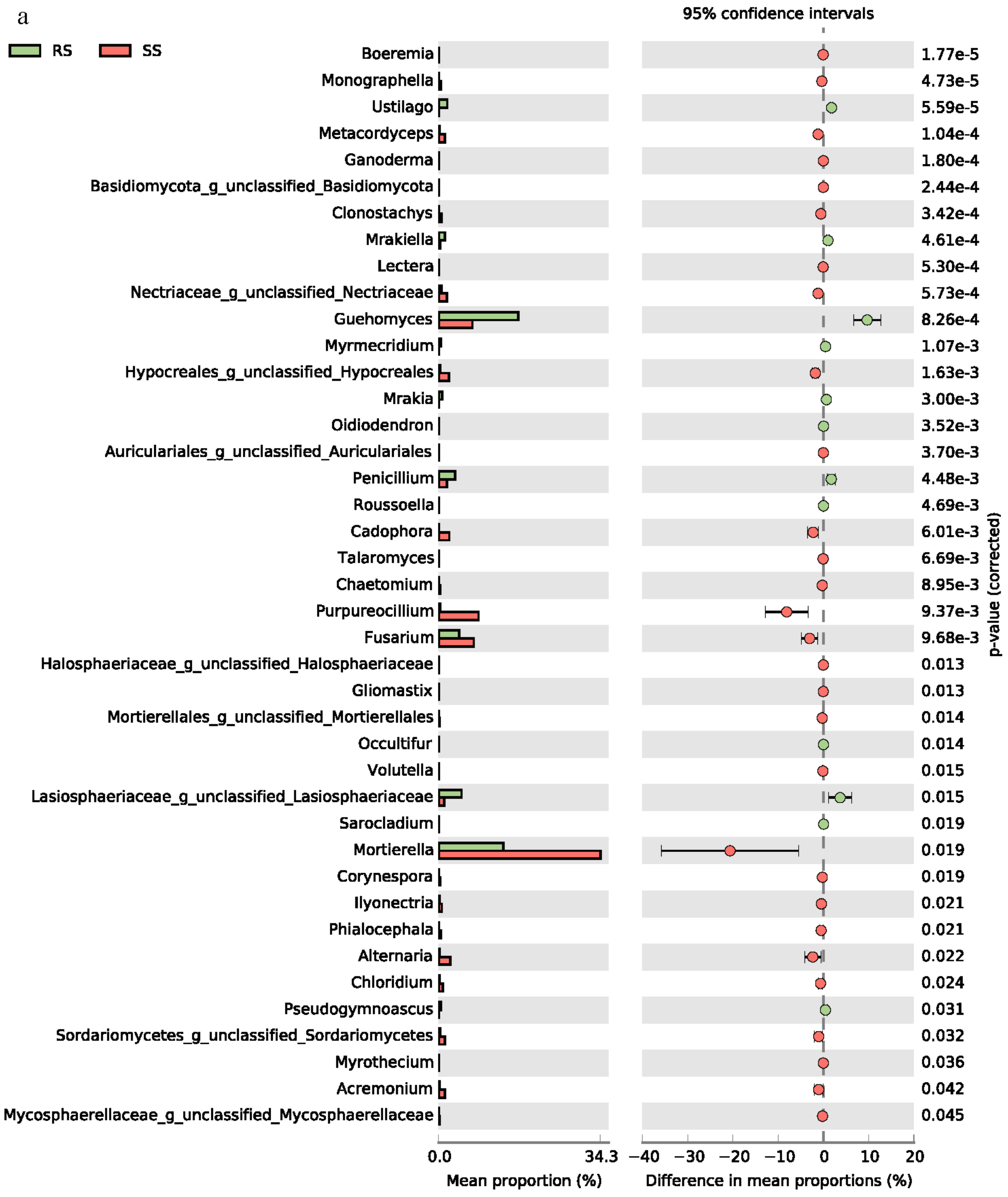
a
RS
SS
95% confidence intervals
Boeremia
Monographella
Ustilago
Metacordyceps
Ganoderma
Basidiomycota_g_unclassified_Basidiomycota
Clonostachys
Mrakiella
Lectera
Nectriaceae_g_unclassified_Nectriaceae
Guehomyces
Myrmecridium
Hypocreales_g_unclassified_Hypocreales
Mrakia
Oidiodendron
Auriculariales_g_unclassified_Auriculariales
Penicillium
Roussoella
Cadophora
Talaromyces
Chaetomium
Purpureocillium
Fusarium
Halosphaeriaceae_g_unclassified_Halosphaeriaceae
Gliomastix
Mortierellales_g_unclassified_Mortierellales
Occultifur
Volutella
Lasiosphaeriaceae_g_unclassified_Lasiosphaeriaceae
Sarocladium
Mortierella
Corynespora
Ilyonectria
Phialocephala
Alternaria
Chloridium
Pseudogymnoascus
Sordariomycetes_g_unclassified_Sordariomycetes
Myrothecium
Acremonium
Mycosphaerellaceae_g_unclassified_Mycosphaerellaceae
1.77e-5
4.73e-5
5.59e-5
1.04e-4
1.80e-4
2.44e-4
3.42e-4
4.61e-4
5.30e-4
5.73e-4
8.26e-4
1.07e-3
1.63e-3
3.00e-3
3.52e-3
3.70e-3
4.48e-3
4.69e-3
6.01e-3
6.69e-3
8.95e-3
9.37e-3
9.68e-3
0.013
0.013
0.014
0.014
0.015
0.015
0.019
0.019
0.019
0.021
0.021
0.022
0.024
0.031
0.032
0.036
0.042
0.045
p-value (corrected)
0.0
34.3
Mean proportion (%)
−40
−30
−20
−10
0
10
20
Difference in mean proportions (%)

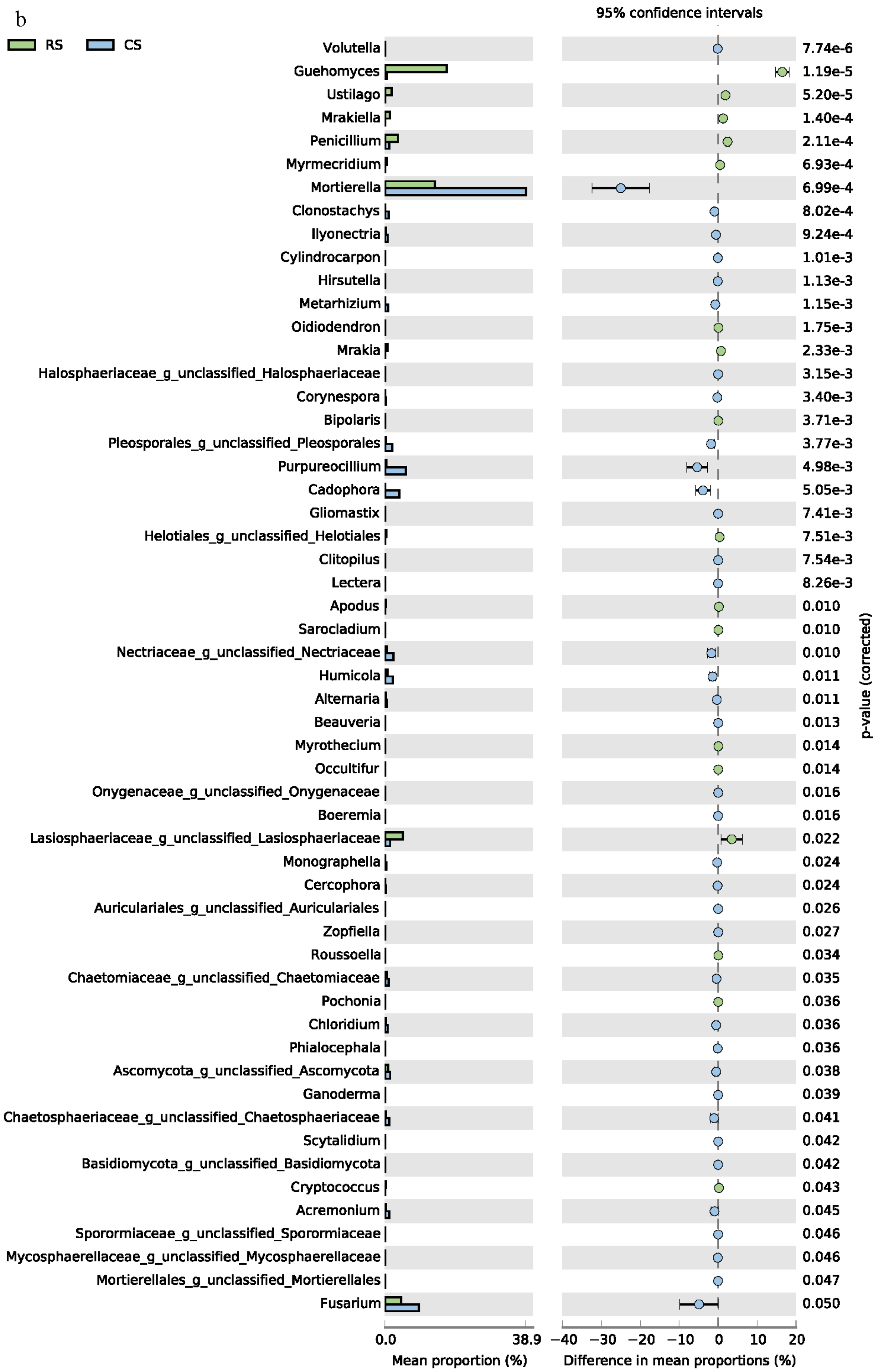

Figure S3 | The variance of relative abundance of fungal genera in response to SS compared with RS (a) and CS compared with RS (b) at 95% confidence intervals.
